# Platelets prime hematopoietic–vascular niche to drive angiocrine-mediated liver regeneration

**DOI:** 10.1038/sigtrans.2016.44

**Published:** 2017-02-17

**Authors:** Koji Shido, Deebly Chavez, Zhongwei Cao, Jane L Ko, Shahin Rafii, Bi-Sen Ding

**Affiliations:** 1 Division of Regenerative Medicine, Department of Medicine, Ansary Stem Cell Institute, Weill Cornell Medicine, New York, New York, USA; 2 Department of Biological Sciences, Seton Hall University, South Orange, New Jersey, USA

## Abstract

In mammals, the livers regenerate after chemical injury or resection of hepatic lobe by hepatectomy. How liver regeneration is initiated after mass loss remains to be defined. Here we report that following liver injury, activated platelets deploy SDF-1 and VEGF-A to stimulate CXCR7^+^ liver sinusoidal endothelial cell (LSEC) and VEGFR1^+^ myeloid cell, orchestrating hepatic regeneration. After carbon tetrachloride injection or hepatectomy, platelets and CD11b^+^VEGFR1^+^ myeloid cells were recruited to LSECs, and liver regeneration in both models was impaired in thrombopoietin-deficient (*Thpo*^−/−^) mice repressing production of circulating platelets. This impeded regeneration phenotype was recapitulated in mice with either conditional ablation of *Cxcr7* in LSEC (*Cxcr7*^iΔ/iΔ^) or *Vegfr1* in myeloid cell (*Vegfr1*^lysM/lysM^). Both *Vegfr1*^lysM/lysM^ and *Cxcr7*^iΔ/iΔ^ mice exhibited suppressed expression of hepatocyte growth factor and Wnt2, two crucial trophogenic angiocrine factors instigating hepatocyte propagation. Of note, administration of recombinant thrombopoietin restored the prohibited liver regeneration in the tested genetic models. As such, our data suggest that platelets and myeloid cells jointly activate the vascular niche to produce pro-regenerative endothelial paracrine/angiocrine factors. Modulating this ‘hematopoietic–vascular niche’ might help to develop regenerative therapy strategy for hepatic disorders.

## Introduction

In mammals, the liver can undergo regeneration after either chemical injury or surgical resection of liver mass, a partial hepatectomy (PH) procedure.^1–10^ This regeneration process is governed by dynamic interplay between parenchymal hepatocytes and non-parenchymal cells (NPCs),^1,2,4,11–14^ including stellate cells,^5,10^ liver sinusoidal endothelial cells (LSECs),^15–23^ biliary epithelial cells^24^ and hematopoietic cells.^25–29^ As such, defining the multicellular interaction orchestrating liver regeneration might help to design therapeutic interventions for hepatic diseases.

LSECs lining hepatic sinusoidal vasculature are essential in choreographing liver organogenesis.^3,15,30–35^ During liver development and regeneration, LSECs produce endothelial-expressed paracrine (angiocrine)^36–41^ cues to regulate synchronized propagation of hepatocyte^12,40^ and resolve fibrosis.^35–39^ However, how angiocrine factor production from LSECs is triggered by liver injury remains to be defined.

Following injury, platelets are recruited to the damaged vasculature. In addition to their hemostatic function, platelets are circulating reservoirs of growth factors.^26,42–47^ We have previously shown that tissue injury triggers secretion of vascular endothelial growth factor-A (VEGF-A) and stromal-derived factor-1 (SDF-1) from platelets.^45^ VEGF-A binds to VEGF receptors 1 and 2 on vascular endothelial cells (ECs)^48,49^ and hematopoietic cells^50–54^ to stimulate production of growth factors. SDF-1 also activates its EC-specific cognate receptor CXCR7 to modulate vascular patterning and angiocrine factor production.^55–57^ The unique function of platelets as major reservoir for VEGF-A and SDF-1 led us to hypothesize that after liver injury, platelets release bioavailable VEGF-A and SDF-1 to prime LSECs and hematopoietic cells, enabling a hematopoietic–vascular niche orchestrating hepatic regeneration.

## Materials and methods

### Animals

C57BL/6J and LysM-Cre mice were obtained from Jackson laboratory. The Chd5(PAC)Cre^ERT2^ mice expressing tamoxifen-responsive Cre^ERT2^ driven by EC-specific VE-cadherin promoter^58,59^ were provided by Dr Ralf Adams. Thrombopoietin (TPO)-deficient (*Thpo*^−/−^) mice^60^ were kindly offered by Dr Frederic J de Sauvage (Genentech, Inc., San Francisco, CA, USA). Mice harboring loxP site-flanked exon 3 of *Cxcr7* (Cxcr7^LoxP/LoxP^) were kindly provided by ChemoCentryx, Inc. (Mountain View, CA, USA). Floxed *Vegfr1* mice were kindly provided by Dr Guo-Hua Fong. Rosa-Cre^ERT2^ animals expressing tamoxifen-responsive inducible Cre were described previously.^15,39^ The Chd5(PAC)Cre^ERT2^ mouse line was crossed with floxed *Cxcr7* mice to generate *Cxcr7*^iΔEC/iΔEC^ mice and control *Cxcr7*^iΔEC/+^ mice after treatment of tamoxifen at a dose of 250 mg kg^−1^ for 6 days, and interrupted for 3 days after the third dose. Mice were rested for at least 20 days after the last injection. *Cxcr7*^LoxP/LoxP^ mice were also crossed with Rosa-Cre^ERT2^ mice to generate Cre^+^*Cxcr7*^loxP/loxP^ mice, resulting *Cxcr7* deletion in adult mice (*Cxcr7*^iΔ/iΔ^). Floxed *Vegfr1* mice were bred with LysM-driven Cre (Jackson, Bar Harbor, ME, USA) to generate mice lacking *Vegfr1* in myeloid cells (*Vegfr1*^lyzM/lyzM^). Deletion of target genes was corroborated by quantitative PCR. Investigators who performed mouse experiments and who analyzed the pattern of cell distribution were randomly assigned with samples, and they were blinded to the genotype of the animals or samples from various groups. All animal experiments were carried out following the guidelines of Institutional Animal Care and Use Committee at Weill Cornell Medicine.

### Mouse liver regeneration and repair models

In all, 70% PH model was used as previously described.^15^ In brief, three most anterior lobes were resected without injuring the blood supply to the caudate and the right lobes after mice were anesthetized by 100 mg kg^−1^ intraperitoneal (i.p.) ketamine and 10 mg kg^−1^ xylazine followed by midline laparotomy. To induce liver injury, single injection of carbon tetrachloride (Sigma-Aldrich, St Louis, MO, USA) in oil at a concentration of 40% (0.64 mg ml^−1^) was injected to mice at a dose of 1.6 mg kg^−1^ to induce acute liver injury.^35,61^ Hepatic regeneration was assessed based on the following criteria: liver lobe weight, hepatocyte proliferation, alanine aminotransferase level and histology using hematoxylin and eosin staining. Six- to ten-week-old mice were utilized and compared.

### Stimulation of thrombopoiesis

To stimulate thrombopoiesis, recombinant TPO, VEGF-A and/or SDF-1 (PeproTech, Rocky Hill, NJ, USA) was injected into *Thpo*^−/−^ or *WT* mice i.p. at a dose of 25 μg kg^−1^ on a daily basis 10 days before PH or CCL_4_ injury and afterwards. Vehicle for individual cytokines was also injected as a control group. The degree of hepatic regeneration was evaluated with control group, including alteration in circulating platelets and parameters of hepatogenesis.

### Immunostaining and morphometric analysis

Tissues were collected and cryopreserved as described^15,35^ for morphometric analysis.^15,36^ Mouse liver was fixed with 4% paraformaldehyde and cryopreserved in optimal cutting temperature compound. For immunofluorescent microscopy, the liver sections (10 μm) were blocked (5% donkey serum/0.3% Triton X-100) and incubated with anti-VE-cadherin (2 μg ml^−1^, R&D Systems, NE Minneapolis, MN, USA) and VEGFR3 (Eli Lilly and Co., New York, NY, USA) antibodies to identify LSECs.^15,45^ To reveal platelet activation, antibodies against CD41 and P-selectin (BD Biosciences, San Jose, CA, USA) were used, respectively. CD11b (BD Biosciences) and VEGFR1 antibodies (Eli Lilly and Co.) were used to detect myeloid cells.^15,45,62^ Id1 staining was performed with rabbit anti-human Id1 antibody (Biocheck, Foster City, CA, USA). After incubation in fluorophore-conjugated secondary antibodies (2.5 μg ml^−1^, Jackson ImmunoResearch, West Grove, PA, USA), sections were counterstained with 4,6-diamidino-2-phenylindole (Invitrogen, Carlsbad, CA, USA). No appreciable staining was observed in isotype IgG controls.

Cell proliferation *in vivo* was measured by 5-Bromo-2′-deoxyuridine (BrdU) uptake. Single dose of BrdU (Sigma, St Louis, MO, USA) at 50 mg kg^−1^ was i.p. injected to mice 1 h before killing. Liver lobes were removed, weighed and slice of tissues were incubated with 1 m HCl at room temperature for 1 h, neutralized with 10 mm Tris (pH 8.5) for 15 min. After incubation with secondary antibody (Jackson ImmunoResearch), cells incorporated with BrdU were identified as proliferating hepatocytes.

### Image acquisition and analysis

Histology analysis of liver sections was captured with Olympus BX51 microscope (Olympus America, Center Valley, PA, USA), and fluorescent images were recorded on AxioVert LSM710 (Carl-Zeiss, Thornwood, NY, USA) confocal microscope. Fluorescent signals in slide were independently evaluated by two investigators from randomly selected fields of view. Parameters from each individual animal were measured and averaged.

### Isolation and cultivation of LSECs

Mouse LSECs were isolated by previously described two-step collagenase perfusion technique with modifications.^15^ In brief, the liver was perfused with Liver Perfusion Medium (Invitrogen), and dissociated by Liver Digest Medium (Invitrogen). The NPCs were fractionated with Percoll (GE Healthcare Bio-Sciences, Pittsburgh, PA, USA) gradient centrifugation with 75% stock Percoll solution and 35% stock Percoll solution. LSEC faction was isolated by mouse LSEC-binding magnetic beads (Miltenyi, Auburn, CA, USA) and Dynabeads Magnetic Beads conjugated with anti-mouse CD31 antibody (MEC13.3, BD Biosciences). Expression of Id1, CXCR7, HGF and Wnt2 messenger RNA was determined. Primary human LSECs were procured from ScienCell Research Laboratories (catalog no. 5000, Carlsbad, CA, USA). Expression of factor VIII was validated by immunostaining. Akt-LSECs were derived from isolated LSECs that were transfected with the pCCL. PGK lentiviral vector with mouse constitutively active Akt1 (myristoylated Akt: myrAkt).^63^ After starving in serum-free medium, 500 000 LSECs were seeded and stimulated with 10 ng ml^−1^ SDF-1. LSECs were also treated with 30 μm Wortmannin (Sigma-Aldrich).

### Flow cytometry analysis

Flow cytometry analysis of platelets and LSECs on isolated liver NPCs as previously described.^15,45,62^ In brief, retrieved livers from killed animals were minced, digested in liver digestion medium (Invitrogen), and filtered through a 30-μm strainer. Single-cell suspensions were preincubated with Fc block (CD16/CD32; BD Biosciences) and then incubated with primary antibodies recognizing mouse LSECs and hematopoietic cells, as well as rat IgG2aκ and IgG2aβ isotype control. Primary antibodies were directly conjugated to different Alexa Fluor dyes or Quantum Dots (BD Biosciences) using antibody labeling kits (Invitrogen). Labeled cell populations were measured by a LSRII flow cytometer (Becton Dickinson, Franklin Lakes, NJ, USA). Compensation for multivariate experiments was carried out with FACS Diva software (Becton Dickinson Immunocytometry Systems, Franklin Lakes, NJ, USA).

### Gene expression analysis by real-time PCR

Total RNA was extracted using RNeasy kit (Qiagen, Germantown, MD, USA). After isolation, 500 ng of total RNA was transcribed into complementary DNA by using the superscript reverse transcriptase kit (Invitrogen). The detection of complementary DNA expression for the specific genes was performed by using the SYBR Green quantitative PCR (Applied Biosystems, Foster City, CA, USA). To selectively knockdown *Cxcr7* in LSECs, shRNA lentiviruses were generated by cotransfecting 15 μg of shuttle lentiviral vector, 3 μg of pENV/VSV-G, 5 μg of pRRE and 2.5 μg of pRSV-REV in 293T cells.^35^ Viral supernatants were concentrated by ultracentrifugation and used to transduce LSECs.

### Statistical analysis

All data were presented as the mean±s.e.m. Comparisons between different groups were made using one-way analysis of variance. Statistical significance was set at *P*<0.05. Each experiment was at least three times.

## Results

**Figure 1:**
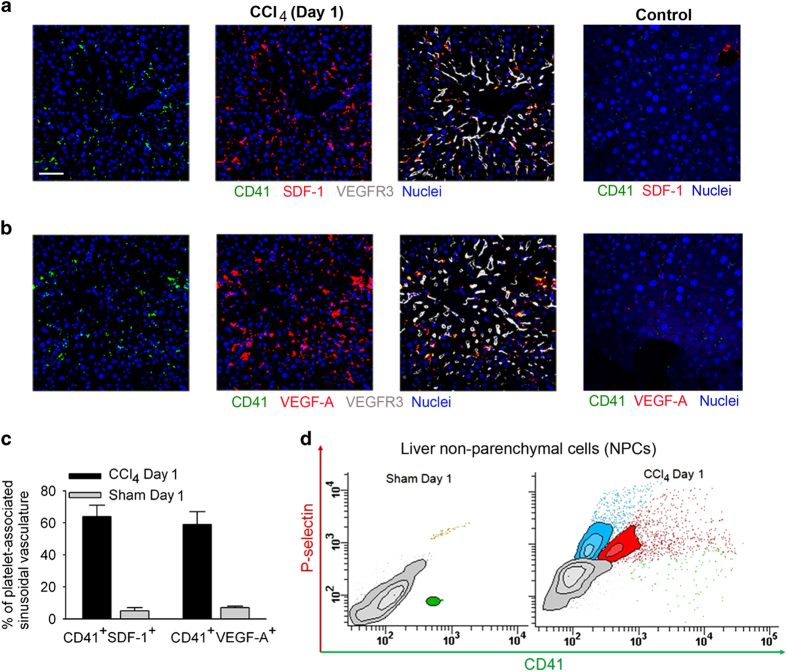
Hepatotoxic injury recruits CD41^+^ platelets carrying VEGF-A and SDF-1 to liver vasculature. (**a**, **b**) After intraperitoneal (i.p.) injection of hepatotoxic agent carbon tetrachloride (CCl_4_), CD41^+^ platelets expressing VEGF-A and SDF-1 were recruited to VEGFR3^+^ liver sinusoidal endothelial cells (LSECs). Liver sections were stained with antibodies against platelet marker CD41, VEGF-A (**a**), SDF-1 (**b**) and LSEC-specific marker VEGFR3. After CCl_4_ injury, but not administration of PBS (control), CD41^+^SDF-1^+^VEGF-A^+^ platelets were associated with VEGFR3^+^ LSECs; scale bar, 50 μm. (**c**) Quantification of CD41^+^SDF-1^+^VEGF-A^+^ platelets associated with VEGFR3^+^ LSECs. *N*=5–7 mice per group. (**d**) Flow cytometry analysis of platelet accumulation in the CCl_4_-injured and control livers. Activation of CD41^+^ platelets was evidenced by surface expression of activation marker P-selectin.

We first examined the localization of platelet-derived VEGF-A and SDF-1 after carbon tetrachloride (CCl_4_)-induced liver injury. VEGF-A and SDF-1 were co-stained with platelet surface marker CD41 in the liver 24 h after CCl_4_ i.p. injection (Figures 1a and b). Compared to sham mice, CCl_4_ injection caused significant deposition of CD41^+^ platelets on VEGFR3^+^ LSECs, with the majority of them stained for VEGF-A and SDF-1 (Figure 1c). Flow cytometric analysis of hepatic NPCs showed that CD41^+^ platelets constituted 24% of NPCs in CCl_4_-injured but not sham mice (Figure 1d), and platelet activation marker, P-selectin, was presented on the surface of 73% of CD41^+^ platelets in the damaged liver. Thus, CCl_4_ injury caused recruitment and local activation of platelets secreting VEGF-A and SDF-1, which might activate hematopoietic and LSECs via VEGF-A and SDF-1 receptors.

**Figure 2:**
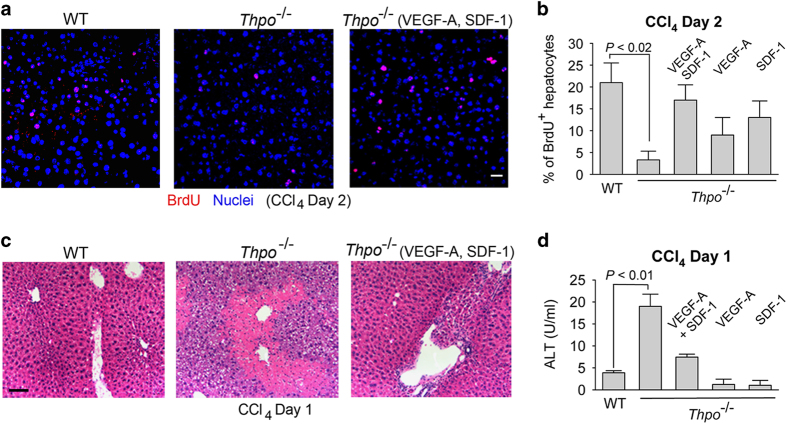
Platelet-deficient mice exhibit impaired liver regeneration after CCl_4_ injury. (**a**, **b**) After CCl_4_ injury, proliferation (BrdU^+^) of hepatocytes was prohibited in thrombopoietin knockout mice (*Thpo*^−/−^) lacking platelets. Hepatocyte proliferation was enhanced in *Thpo*^−/−^ mice by injection of VEGF-A and SDF-1. *N*=6–8 mice per group. (**c**) Severe centrilobular damage in the liver of *Thpo*^−/−^ mice after CCl_4_ injury, as indicated by scattered cell debris in *Thpo*^−/−^ mice relative to mild centrilobular necrosis in *WT* mice. VEGF-A and SDF-1 injection ameliorated the injury in *Thpo*^−/−^ mice. Scale bar, 50 μm. (**d**) Increased hepatic injury (plasma alanine aminotransferase, ALT activity) in *Thpo*^−/−^ mice following CCl_4_ injection, as compared to *WT* mice. These data imply that VEGF-A and SDF-1 produced by activated platelets protect against acute hepatotoxic injury. *N*=6–8 mice per group.

To test the contribution of platelets in protecting against liver injury, we examined mice deficient of TPO (*Thpo*^−/−^) after CCl_4_ injection. Platelet number is decreased in *Thpo*^−/−^ mice by 95% as compared to wild-type (*WT*) mice.^60,64^ Hepatocyte proliferation after CCl_4_ injection was significantly reduced in *Thpo*^−/−^ mice than that of *WT* control group (Figures 2a–c). Meanwhile, hepatic injury was markedly increased, as indicated by elevation of plasma alanine aminotransferase activity (Figure 2d). Of note, the impaired hepatocyte proliferation and enhanced hepatic injury in *Thpo*^−/−^ mice were rescued by injection of VEGF-A and/or SDF-1 (Figures 2a–d). Therefore, activated platelets recruited to the injured vascular bed supply SDF-1 and VEGF-A to stimulate hepatic repair.

**Figure 3:**
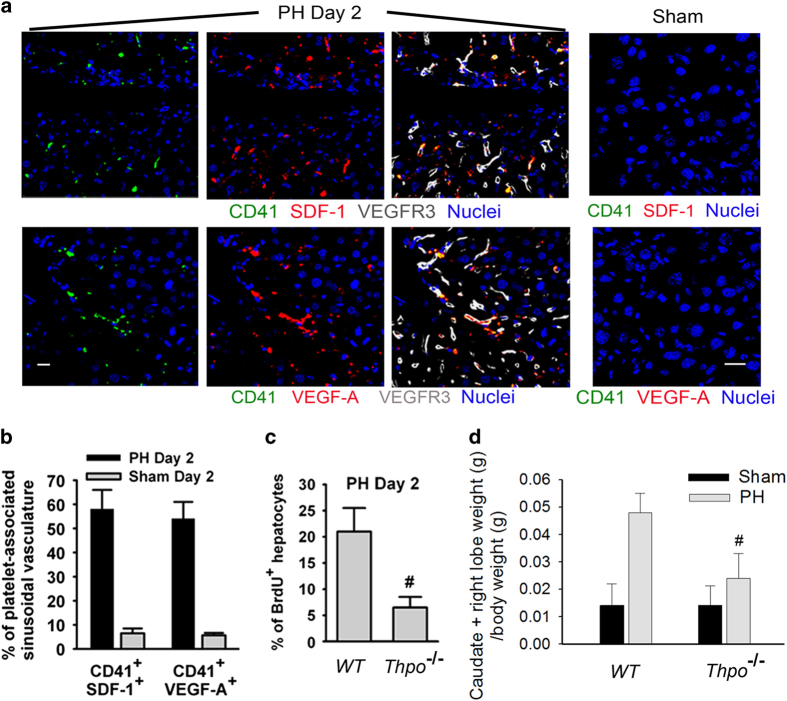
After 70% partial hepatectomy (PH), platelets harboring VEGF-A and SDF-1 are associated with VEGFR3^+^ LSECs. (**a**, **b**) Liver sections were stained with antibodies to CD41 (green), VEGF-A and SDF-1 (red), and VEGFR3 (gray). After PH, but not sham operation (Sham), CD41^+^SDF-1^+^VEGF-A^+^ platelets are associated with VEGFR3^+^ LSECs. Scale bar, 50 μm. (**c**, **d**) BrdU incorporation in hepatocyte (**c**) and liver mass restoration (**d**) in *Thpo*^−/−^ and WT mice at days 2 and 8 after PH, respectively. ^#^*P*<0.05, compared to control group; *N*=5–8 mice per group.

We then further examined the contribution of platelets to liver regeneration after 70% PH. Two days after PH, CD41^+^ platelets were similarly recruited onto VEGFR3^+^ LSECs (Figure 3a). There was a co-localization of SDF-1 and VEGF-A with CD41^+^ platelets on the surface of VEGFR3^+^ LSECs (Figures 3a and b). Proliferation of hepatocytes and liver mass restoration after PH were diminished in *Thpo*^−/−^ mice relative to *WT* group (Figures 3c and d). These data implicate that platelets produce VEGF-A and SDF-1 to prime LSECs, eliciting liver regeneration.

**Figure 4:**
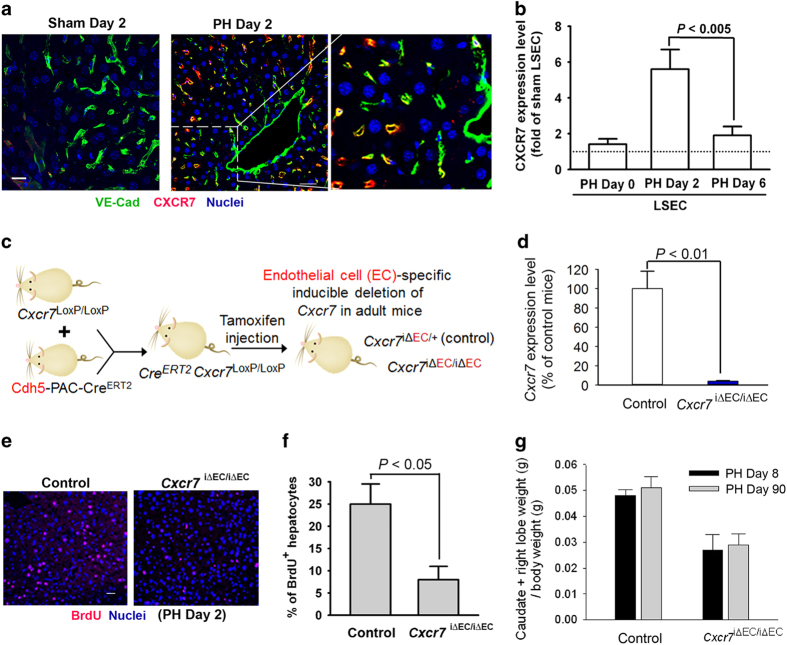
After PH, SDF-1 receptor CXCR7 is upregulated in LSEC and contributes to hepatocyte proliferation. (**a**) Two days after PH, the liver sections were stained for endothelial-specific VE-cadherin (green fluorescence). VE-cadherin^+^ LSECs are co-localized with CXCR7^+^ LSECs (red fluorescence). Scale bar, 50 μm. (**b**) *Cxcr7* mRNA level in isolated LSECs was examined at indicated time after PH. The *Cxcr7* expression level of sham LSEC was arbitrarily defined as 1. *N*=5–7 mice per group. (**c**, **d**) Mice harboring loxP-flanked *Cxcr7* were crossed with endothelial cell-specific Cdh5-PAC-Cre^ERT2^ mice.^58^ Generated offsprings were treated six times with tamoxifen injection (250 mg kg^−1^) to induce *Cxcr7* deletion (*Cxcr7*^iΔEC/iΔEC^).^15^ Mice carrying endothelial haplodeficiency of *Cxcr7* (*Cxcr7*^iΔEC/+^) were used as control group. *N*=4 mice per group. (**e**–**g**) Inducible knockout of *Cxcr7* in LSEC abrogated hepatocyte proliferation (**e**, **f**) and restoration of liver mass at indicated time after PH (**g**). Prohibition of liver mass recovery in *Cxcr7*^iΔEC/iΔEC^ mice persisted for up to 90 days after PH; *N*=6–8 mice per group, *P*<0.01 between control and *Cxcr7*^iΔEC/iΔEC^ group.

SDF-1 confers its pro-angiogenic activity^65^ through the activation of two receptors, CXCR4 and CXCR7. CXCR7 expression is mainly enriched in ECs and subsets of lymphocytes,^55,57,66,67^ and CXCR7 activation in EC is essential for the production of pro-regenerative angiocrine factor in organ repair.^35,39^ Therefore, we examined the expression pattern and functional attributes of EC-specific SDF-1 receptor, CXCR7, after PH (Figure 4a). Immunostaining shows that the expression of CXCR7 was upregulated in LSEC 2 days post PH, compared to sham-operated mice (Figures 4a and b). Therefore, CXCR7 might serve as an inducible LSEC-specific SDF-1 receptor after acute liver injury.

To test functional contribution of CXCR7 in liver regeneration, we selectively deleted *Cxcr7* in ECs of adult mice using an inducible tamoxifen-responsive Cre^ERT2^ that is specifically expressed in ECs^58^ (Figure 4c). Mice expressing floxed *Cxcr7* were bred with mouse line carrying EC-specific VE-Cadherin-Cre^ERT2^/Cdh5(PAC)Cre^ERT2^.^58^ I.p. injection of tamoxifen to resulting offsprings induced 96% of *Cxcr7* deletion in ECs of adult mice (Figure 4d). These mice lacking *Cxcr7* in ECs (*Cxcr7*^iΔEC/iΔEC^) were subjected to PH, and liver regeneration was compared with control mice harboring endothelial haplodeficiency of *Cxcr7* (*Cxcr7*^iΔEC/+^). Hepatocyte proliferation and liver mass regeneration were markedly reduced in *Cxcr7*^iΔEC/iΔEC^ mice, as compared to those of control mice (Figures 4e–g). Thus, endothelial CXCR7 might be essential for promoting liver regeneration after PH.

**Figure 5:**
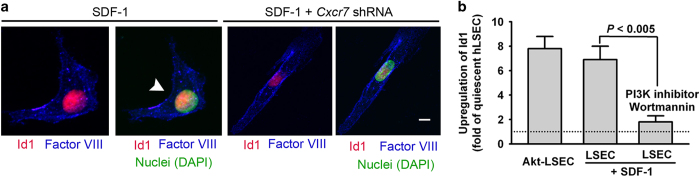
SDF-1 activates CXCR7 on LSEC, inducing Akt-dependent activation of Id1 angiocrine pathway. (**a**) CXCR7 expression is essential for SDF-1-mediated Id1 nuclear induction. Early passages of primary human factor VIII^+^ LSECs (hLSECs) were stimulated with 10 ng ml^−1^ of SDF-1. shRNA silencing of *Cxcr7* in human LSEC abolished nuclear accumulation of Id1 after SDF-1 treatment. Note SDF-1-induced translocation of Id1 from the cytoplasm to nuclei (arrow head). Scale bar, 10 μm. (**b**) PI3 kinase inhibitor Wortmannin blocked SDF-1-mediated upregulation of Id1, implicating that SDF-1 induction of Id1 is Akt-dependent. *N*=3–5 independent experiments.

After PH, LSECs produce hepatic-active paracrine/angiocrine growth factors such as HGF and Wnt2.^15^ This angiocrine function of LSECs in liver regeneration depends on the activation of transcription factor inhibitor of DNA binding 1 (Id1) in LSEC.^15,37^ We then assessed the effect of SDF-1 on cultured human LSECs. SDF-1 induced both upregulation and nuclear enrichment of Id1 protein in LSEC, which was abrogated by genetic silencing of *Cxcr7* (Figures 5a and b). In addition, SDF-1-dependent Id1 upregulation in LSEC was recapitulated by Akt overexpression and suppressed by Wortmannin, an inhibitor of PI3 kinase–Akt pathway (Figure 5b). These data imply that SDF-1 stimulates CXCR7 in LSEC to trigger Akt-dependent activation of Id1 angiocrine pathway.

**Figure 6:**
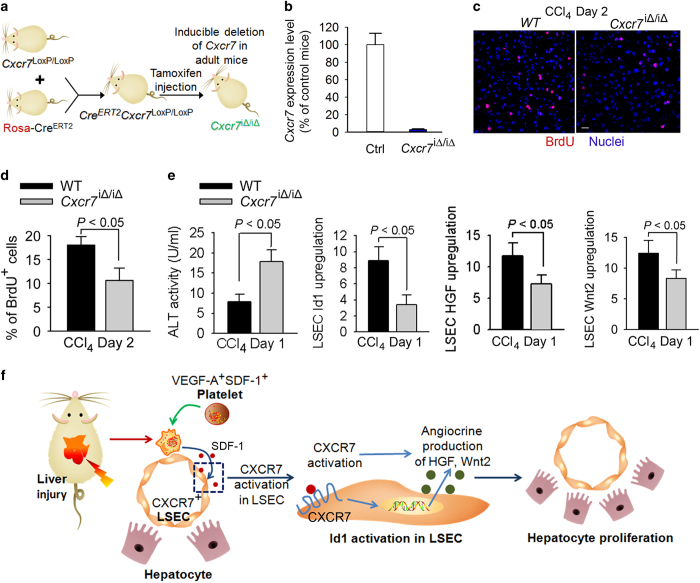
After CCl_4_-induced liver injury, CXCR7 stimulates angiocrine-mediated hepatic regeneration. (**a**, **b**) Mice harboring loxP site-flanked *Cxcr7* were crossed with Rosa-Cre^ERT2^ and treated six times with tamoxifen injection (250 mg kg^−1^) to induce the deletion of *Cxcr7* in adult mice (*Cxcr7*^iΔ/iΔ^).^15^ Transcriptional level of *Cxcr7* in the liver is shown in **b**; *N*=6–9 mice per group. (**c**, **d**) Inhibition of SDF-1 signaling in LSEC abolished liver regeneration after CCl_4_ injury. In *Cxcr7*^iΔ/iΔ^ mice, the decrease in cell proliferation was determined by staining for BrdU incorporation; *N*=6 mice per group. Scale bar, 50 μm. (**e**) Increased hepatic damage in *Cxcr7*^iΔ/iΔ^ mice after CCl_4_ injury was associated with abrogated induction of Id1-Wnt2/hepatocyte growth factor (HGF) angiocrine pathway; *N*=5 mice per group. (**f**) Platelet-dependent activation of LSECs leads to the generation of hepatogenic angiocrine factors. Upon hepatotoxic injury, activated platelets generate SDF-1 to turn on Id1 pathway in LSECs, resulting in angiocrine production of HGF and Wnt2 that initiates hepatocyte propagation and liver repair.

We then assessed whether CXCR7 is responsible for inducing Id1-dependent production of angiocrine factors for liver repair. Given that CXCR7 is specifically upregulated in LSECs after liver injury,^36^ we utilized a tamoxifen-inducible Rosa-Cre^ERT2^ system to ablate *Cxcr7* in adult mice (Figure 6a). *Cxcr7*^LoxP/LoxP^ mice were crossed with Rosa-Cre^ERT2^ mice to generate Cre^ERT2+^*Cxcr7*^loxP/loxP^ mice. Injection of tamoxifen induced deletion of *Cxcr7* in adult mice (*Cxcr7*^iΔ/iΔ^; Figure 6b), and liver regeneration was compared between *WT* and *Cxcr7*^iΔ/iΔ^ mice after CCl_4_ injection. There were significantly lower extent of hepatocyte proliferation (BrdU incorporation) and higher degree of hepatic injury in *Cxcr7*^iΔ/iΔ^ mice than those of *WT* mice (Figures 6c and d). Activation of Id1-HGF/Wnt2 angiocrine pathway was markedly prohibited in *Cxcr7*^iΔ/iΔ^ mice, as compared to *WT* mice (Figure 6e). These results suggest that after liver injury, platelets supply SDF-1 to activate CXCR7^+^ LSECs, inducing the production of pro-regenerative angiocrine Wnt2 and HGF for hepatic repair (Figure 6f).

**Figure 7:**
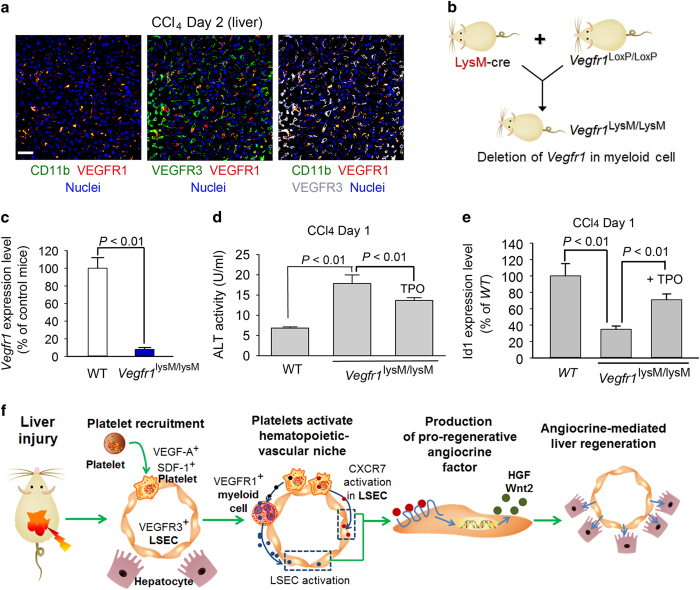
Activated platelets recruit VEGFR1^+^ myeloid cells to reinforce angiocrine-mediated liver repair. (**a**) Recruited VEGFR1^+^ cells in the liver co-expressed CD11b and were associated with VEGFR3^+^ LSECs, as revealed by immunostaining. Scale bar, 50 μm. (**b**, **c**) Conditional knockout of *Vegfr1* in myeloid cells abrogated hepatocyte proliferation after CCl_4_ injection. Floxed *Vegfr1* mice were bred with LysM-driven Cre to generate mice lacking *Vegfr1* in myeloid cells (*Vegfr1*^lyzM/lyzM^). Quantification of *Vegfr1* transcriptional level in myeloid cells is shown in **c**; *N*=5 mice per group. (**d**) Exacerbated liver injury in *Vegfr1*^lyzM/lyzM^ mice than *WT* control, as evidenced by elevated plasma level of alanine aminotransferase, ALT. Injection of thrombopoietin (+TPO) prevented liver parenchymal injury in *Vegfr1*^lyzM/lyzM^ mice; *N*=5–7 mice per group. (**e**) Pro-regenerative Id1 angiocrine pathway was suppressed in *Vegfr1*^lyzM/lyzM^ mice, which was elevated by thrombopoietin injection (+TPO). Compared to *WT* control mice, transcriptional level of Id1 was lower in *Vegfr1*^lyzM/lyzM^ mice after CCl_4_ injury. *P<0.05, compared to *Vegfr1*^lyzM/lyzM^ group. *N*=5–7 mice per group. (**f**) Schema depicting the contribution of hematopoietic–vascular niche for liver regeneration. Upon liver injury, activated platelets are recruited to the liver and produce SDF-1 to activate CXCR7^+^ LSECs, initiating endothelial paracrine/angiocrine-mediated liver repair. Activation of VEGFR1^+^ myeloid cells by platelet VEGF-A further stimulate Id1-Wnt2/HGF angiocrine pathway in LSEC, reinforcing liver regeneration.

In the CCl_4_-injured liver, platelets carry both SDF-1 and VEGF-A. Hence, we investigated the contribution of VEGF-A receptors in mediating liver repair. VEGFR1 activation mediates early protection after CCl_4_ liver injury.^16,68^ Thus, we tested the contribution of VEGFR1-expressing cells in the injured liver.^54^ CCl_4_ injection caused significant recruitment of VEGFR1^+^CD11b^+^ myeloid cells to the liver, adhering to VEGFR3^+^ LSECs (Figure 7a). To determine the contribution of VEGFR1^+^ myeloid cells to liver repair, *Vegfr1*^loxP/loxP^ mice were crossed with LysM-driven Cre to generate mice lacking *Vegfr1* specifically in myeloid cells (*Vegfr1*^lysM/lysM^) (Figures 7b and c). Liver damage was substantially increased in *Vegfr1*^lysM/lysM^, as evidenced by elevated plasma alanine aminotransferase activity (Figure 7d), and repeated injection of recombinant TPO in *Vegfr1*^lysM/lysM^ mice mitigated the injury. Of note, hepatogenic angiocrine Id1 pathway was suppressed in *Vegfr1*^lysM/lysM^ mice compared to control group, which was restored by TPO injection (Figure 7e). Thus, liver injury recruits platelets and VEGFR1^+^ myeloid cells to jointly activate LSEC niche, driving liver repair (Figure 7f).

## Discussion

LSECs lining hepatic sinusoids actively participate in liver repair and regeneration. Activation of transcription factor Id1 in LSECs leads to the elaboration of hepatic-active angiocrine factors.^15^ In the mouse liver, we have identified a preferential distribution of SDF-1 receptor CXCR7 on LSECs, which was upregulated by PH. We have also revealed the functional role of endothelial CXCR7 in generating hepatic-active factors in both PH and CCl_4_ models. As such, both hepatotoxic injury and loss of liver mass stimulate CXCR7 activation in LSECs, eliciting hepatic regeneration, and repair.

Recruited platelets in the injured liver initiates LSEC angiocrine signaling to trigger hepatic reconstitution. Post injury, platelets serve as circulating sentinel cells to promote tissue repair.^26,42–46^ Here our study has implied a paradigm in which platelet-supplied SDF-1 activates CXCR7 on LSECs and initiates subsequent angiocrine signaling. The beneficial effect of platelets in liver repair is in agreement with both preclinical and clinical findings that favorable prognosis of hepatic function correlates with higher circulating platelet count.^29^ Whether recombinant TPO has the similar protective effect in infectious liver injury remains to be investigated.^47^

The cytoprotective effect of VEGFR1^+^ myeloid cells after acute injury was evidenced by increased injury in mice with myeloid-specific *Vegfr1* knockout (*Vegfr1*^lysM/lysM^). The rescue effect of TPO injection might depend on both platelets and myeloid cells. The effect of VEGFR1^+^ myeloid cells on angiocrine function was evidenced by the diminished Id1 pathway in LSECs of *Vegfr1*^lysM/lysM^ mice. Conceivably, increasing platelet number by TPO injection enhances platelet-dependent activation of myeloid cells, reinforcing endothelial activation and vascular niche-mediated liver regeneration.

The finding that, after liver injury, administration of TPO, SDF-1 and VEGF-A enhanced liver repair has clinical relevance. First, it is conceivable that in thrombocytopenic patients, liver repair is impaired. Thus, cautiously increasing the number of circulating platelet might offer tissue protection. Alternatively, after acute injury, administration of SDF-1 and VEGF-A might augment hepatocyte proliferation by stimulating angiocrine factor generation in LSECs. Furthermore, transfusion/transplantation of properly primed platelets or myeloid cells can possibly offer optimal cell therapy approaches.

Taken together, we demonstrate a pro-regenerative interplay between platelets, myeloid cells and LSECs in liver repair. Platelets play an instrumental role in priming both angiocrine function of LSECs and myeloid cells post injury. Thus, platelet activation enables a hematopoietic–vascular^33^ niche that orchestrates liver regeneration. Identifying critical pathways establishing this hepatogenic hematopoietic–vascular niche might aid in devising regenerative therapy for hepatic diseases.
